# Quantitative electroencephalography interpretation of human brain activity after COVID-19 before and after Sudarshan Kriya Yoga

**DOI:** 10.3389/fnhum.2022.988021

**Published:** 2022-10-07

**Authors:** Marta Kopańska, Barbara Kuduk, Anna Łagowska, Wiktoria Mytych, Renata Muchacka, Agnieszka Banaś-Za̧bczyk

**Affiliations:** ^1^Department of Pathophysiology, Institute of Medical Sciences, Medical College of Rzeszow University, Rzeszow, Poland; ^2^Student's Science Club “Reh-Tech”, University of Rzeszow, Rzeszow, Poland; ^3^Department of Animal Physiology and Toxicology, Pedagogical University of Cracow, Kraków, Poland; ^4^Department of Biology, Institute of Medical Sciences, Medical College of Rzeszow University, Rzeszow, Poland

**Keywords:** QEEG, Sudarshan Kriya Yoga (SKY), respiratory training, COVID-19, mental health care

## Abstract

The COVID-19 pandemic has affected the entire world. The SARS-CoV-2 virus is wreaking havoc globally, leading to serious health problems and even death. The purpose of this study is to present the brainwave variability pattern using QEEG after exposure to COVID-19 and to introduce the subject of the Sudarshan Kriya Yoga (SKY)-based breathing technique. QEEG is one of the basic neurological examinations through which we can compare the changes in the nervous system after SARS-CoV-2 virus infection and observe the variation of brainwave frequencies with a breathing technique.

## Introduction

The SARS-CoV-2 virus has quickly spread across continents, causing the COVID-19 pandemic, officially announced by the World Health Organization (WHO) on 11 March 2020 (Hasöksüz et al., 2020). Infected patients show symptoms of severe respiratory infection, which may lead to acute respiratory distress syndrome (ARDS) and multi-organ complications (Hasöksüz et al., 2020; Wang et al., 2020). Increasingly, post-infection patients report neurological complications and central nervous system symptoms, including encephalitis, taste disorders, anosmia, cerebrovascular disease, toxic encephalopathy, impaired consciousness, problems with concentration, headache, confusion, and dementia (Iadecola et al., 2020; Lopez-Leon et al., 2021; SeyedAlinaghi et al., 2021). Quantitative electroencephalogram (QEEG) is a non-invasive, safe, easy, and effective method of electrophysiological and functional brain imaging. QEEG refers to the quantitative analysis of the EEG recording in which data are digitally encoded and statistically analyzed. In this study, we decided to use the innovative tool of QEEG to distinguish the quantitative distribution of brain waves before, during, and after the Sudarshan Kriya Yoga (SKY) course (Art of Living) in people who had previously had SARS-CoV-2 infections. QEEG enables visual analysis and interpretation of EEG, which significantly broadens the understanding of EEG and brain functioning (Kesebir and Yosmaoglu, 2018; Pastor et al., 2020). The obtained data are characterized by high temporal resolution, contributing to the clinical assessment. It includes affective and cognitive components for assessing the current situation of the disorder, allowing for obtaining information about abnormalities in the functioning of the brain (Kesebir and Yosmaoglu, 2018). Currently, QEEG is one of the main therapeutic methods used in neurological patients with various disorders. With the QEEG technique, it is possible to visualize not only the influence of the SARS-CoV-2 virus on the human nervous system but also the effectiveness and progress of interventions. Therefore, QEEG can also be used in diagnosing and treating patients who develop neurological disorders due to SARS-CoV-2 virus infection (Pastor et al., 2020; Campanella et al., 2021). Information obtained from QEEG can provide the basis for functional assessment of the brain, diagnose disorders, and differentiate their effects (Weon et al., 2021). It is necessary to choose the most individualized therapeutic protocol corresponding to the patient's current state to detect disorders (Kesebir and Yosmaoglu, 2018; Kopańska et al., 2021). By performing precise tests, we can determine the nature of the changes that arise after suffering from COVID-19, whether short-term, chronic, or irreversible (Kopańska et al., 2021; Østergaard, 2021). The breathing-based meditation technique, Sudarshan Kriya Yoga (SKY), involves controlled, rhythmic, and cyclic breathing that helps with problems related to the mind, body, and emotions (Zope and Zope, 2013). The SKY breathing technique can be used to treat anxiety, depression, stress-related illnesses, and posttraumatic stress disorder. A rhythmic breathing form of yoga [hereby mentioned as Sudarshan Kriya Yoga (SKY)] positively affects the nervous system (Vasudev et al., 2020). SKY affects the hypothalamic-pituitary-adrenal (HPA) axis (Zope and Zope, 2013). SKY as a breathing technique may have significant prophylactic and therapeutic potential. Preliminary studies suggest that oxidative defense in the body occurs while practicing SKY, as well as an increase in T and helper T lymphocytes and NK cells, and generally causes relaxation. These phenomena suggest an improvement in the body's resistance to viral infections and an improvement in mental health. One of the non-governmental and non-profit organizations,' Art of Living (AOL), enables people to learn this breathing method, which provides its online breathing and meditation program to millions of people in the COVID-19 pandemic, improving their mental and physical health (Zope et al., 2021). Breathing stimulates the electrical activity of the brain. Numerous scientific studies have established the relationship between the breathing cycle and brain activity (Adrian, 1942; Ito et al., 2014; Heck et al., 2016; Zelano et al., 2016; Herrero et al., 2018). Yoga practices with conscious and controlled variation in breathing frequency increase the synchronization of interhemispheric brainwaves, which improves neurocognitive abilities and emotional states. As a rhythmic breathing technique, Sudarshan Kriya has been shown to reduce stress and anxiety (Kjellgren et al., 2007) and influence autonomic balance (Kharya et al., 2014). Research confirms the effects of SKY on brain activity, leading to better memory, attention, emotional control, and a higher cognitive state. In addition, it improves interhemispheric synchronization and induces resting alertness (Bhaskar et al., 2020). Given the aforementioned scientific reports indicating the negative effects of SARS-CoV-2 virus infection on the nervous system, resulting in conditions such as brain fog, concentration and memory disorders, and the impact of SKY on the immune system and activation in the brainwave area, indicating the improvement of cognitive functions and emotional control, it was decided to investigate whether this particular breathing technique would improve the nervous system disturbances in people who had previously had SARS-CoV-2 virus infection. Confirmation of this premise may be a breakthrough in the treatment of the negative effects of COVID-19 on the human nervous system. Our pilot study aimed to interpret the brainwaves of post-COVID patients before and after Sudarshan Kriya Yoga (SKY).

## Materials and methods

The research was conducted on 15 people. In the private interview questionnaire, they all indicated they had COVID-19. All patients had an antigen test for COVID-19 due to significantly decreased mood and loss of taste. The test was positive in fourteen patients and negative in one patient. Nevertheless, all patients were referred for PCR testing to verify the diagnosis. All the tests were positive. Infection with the virus was moderate in all subjects. All patients lost their taste and smell. Four patients were taking codeine syrup due to the development of a mild cough; the others were not taking any medication. All the probants were 40–50 years old and highly educated. All participants were from the Rzeszow area. Regardless of the coronavirus variant, the respondents had similar disease symptoms, significantly reducing the body's efficiency in all infected people and, thus, declining mental health. Therefore, in our work, we generally pay attention to the variability of the brain wave range under the influence of the SARS-CoV-2 virus, regardless of the type of mutation. We hope our research will inspire future researchers and significantly influence the scope of research, considering various mutations. Approximately 2 months after COVID-19, patients enrolled in the SKY breathing technique program. All patients showed a negative PCR test before the study. Each examination of one person lasted about 15 min and consisted of two stages: the first recording of brain waves with the eyes closed lasted 3 min, and the second one with the eyes open for 3 min. The rest of the time was used for the application and calibration of the equipment and patient instruction. The QEEG examination was carried out before the SKY course (just before the start of the course) and after the SKY course [after the second long SKY, i.e., the third (last) day of the course]. The Art of Living Foundation conducts courses the same way around the world where Sudarshan Kriya Yoga is introduced. The courses are conducted by certified and properly trained teachers. The course currently lasts 3 days, during which the students perform long SKY on the second and third days. On the last day (the third day), students receive the instructions for practicing short (home) SKY daily. Quantitative electroencephalography (QEEG) data were collected by a researcher with Elmiko Board Certification in Neurofeedback. Taking into account the basic principles of QEEG analysis in an adult (at rest and with eyes open and closed), it is assumed that the lower the frequency of the waves, the lower the amplitude (delta < 20 μV, theta in adults < 15 μV, alpha in adults < 10 μV, SMR, beta1, and beta2 within 4–10 μV). The QEEG data were collected by measuring all waves from central points (Cz, C3, and C4), frontal points (Fz, F3, and F4), and parietal points (P3, P4) based on the international 10–20 system. The EEG signal was quantitatively transformed with the Elmiko DigiTrack software (version 14, PL) (ELMIKO, Warsaw, Poland). The conducted studies included delta, theta, alpha, SMR, beta1, and beta2 waves on the Fz, Cz, C3, C4, P3, P4, F3, and F4 electrodes. We considered the Alfa, Beta 1, and Beta2 μV amplitudes (the most important waves for relaxation and concentration) from all points in which there were significant changes. The amplitude of the QEEG rhythms is calculated according to the medical standards of the Digi Track apparatus. The spectrum of a signal is a frequency-dependent representation of this signal. The FFT algorithm is used, with the result of the function: f(z) = A(z) + j^*^F(z). The spectrum analysis results in the FFT panel in DigiTrack show peak-to-peak amplitudes. The epoch length determines the Fourier frequency resolution, with a 1-s epoch providing a 1 Hz resolution (plus/minus 0.5 Hz resolution) and a 4-s epoch providing $\frac{1}{4}$ Hz, or plus/minus 0.125 Hz resolution. The elimination of artifacts from the EEG recording was performed manually and automatically. There were just a few seconds left until the whole examination was over.

### Statistical analyses

The comparison of the values of the quantitative variables at two repeated measurements was performed using the Wilcoxon paired *t*-test. The analysis assumed a significance level of 0.05. Thus, all *p*-values below 0.05 were interpreted as indicative of significant relationships. The analysis was performed in R software, version 4.2.1. R Core Team (2022). (R: A language and environment for statistical computing. R Foundation for Statistical Computing, Vienna, Austria. URL https://www.R-project.org/, available from 10.04.2022).

## Results

### Alpha, open eyes

**Figure 1:**
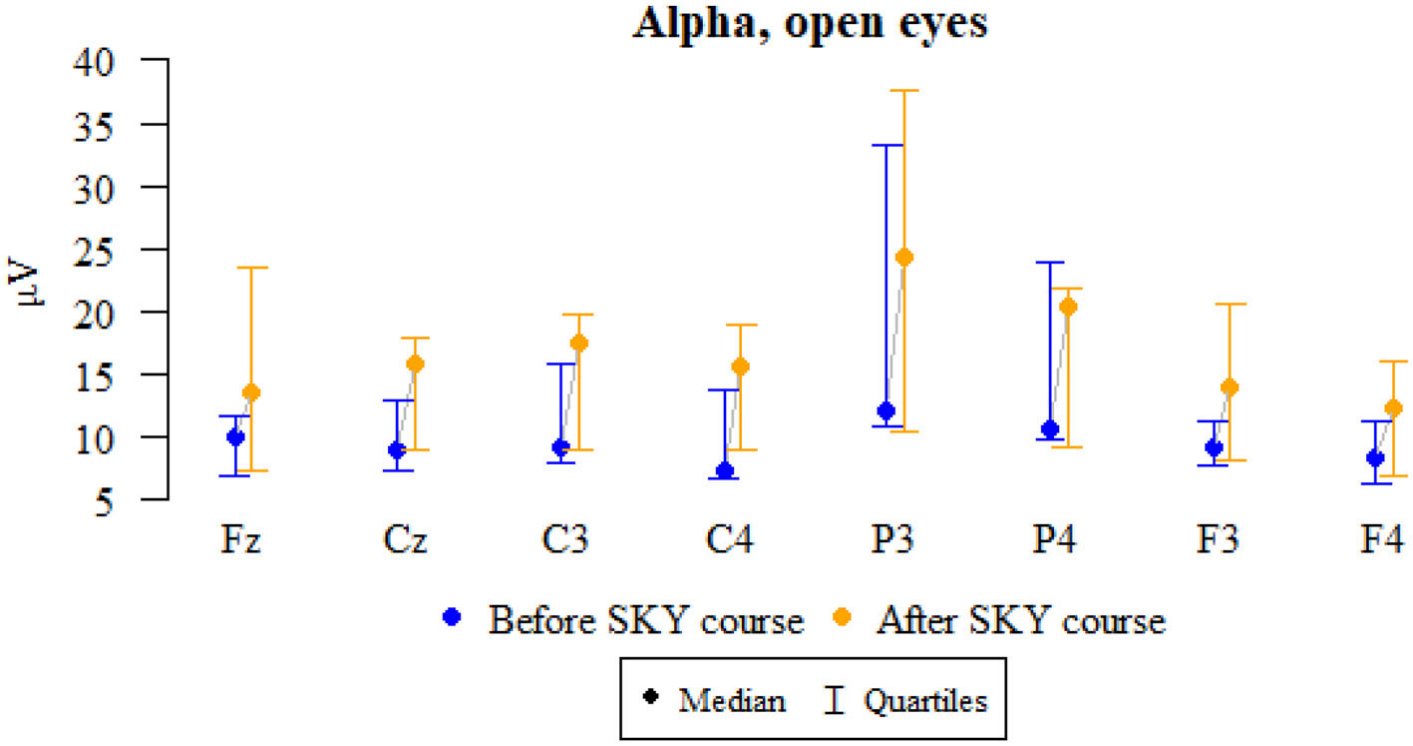
Graphical comparison of Alpha wave amplitudes before the SKY course (the first day) and after the whole SKY course (third day) with eyes open.

Values of *p* < 0.05 indicate statistically significant relationships are as follows:

The amplitudes at Fz, Cz, C3, C4, P3, F3, and F4 increased significantly after relaxation exercises (Figure 1 and Table 1).

**Table 1:** Comparison of Alpha wave amplitudes before the SKY course (first day) and after the whole SKY course (third day) with eyes open.

**Measurement point**	**Measurement**	***N***	**Mean**	**SD**	**Median**	**Min**	**Max**	**Q1**	**Q3**	***p***
Fz	Before relaxation	15	9.54	2.03	10.03	6.95	11.64	6.95	11.64	*p* = 0.002*
	After relaxation	15	14.85	6.99	13.65	7.31	23.58	7.31	23.58	
Cz	Before relaxation	15	9.73	2.48	8.93	7.31	12.96	7.31	12.96	*p* = 0.002*
	After relaxation	15	14.31	4.00	15.89	9.03	18.01	9.03	18.01	
C3	Before relaxation	15	11.04	3.64	9.30	7.92	15.91	7.92	15.91	*p* = 0.002*
	After relaxation	15	15.36	4.84	17.44	8.94	19.70	8.94	19.70	
C4	Before relaxation	15	9.27	3.29	7.30	6.80	13.72	6.80	13.72	*p* = 0.002*
	After relaxation	15	14.59	4.30	15.67	9.09	19.00	9.09	19.00	
P3	Before relaxation	15	18.80	10.68	12.19	10.96	33.24	10.96	33.24	*p* = 0.024*
	After relaxation	15	24.17	11.60	24.43	10.44	37.63	10.44	37.63	
P4	Before relaxation	15	14.79	6.71	10.74	9.77	23.86	9.77	23.86	*p* = 0.843
	After relaxation	15	17.17	5.98	20.46	9.12	21.94	9.12	21.94	
F3	Before relaxation	15	9.46	1.59	9.31	7.67	11.39	7.67	11.39	*p* = 0.002*
	After relaxation	15	14.26	5.32	13.88	8.22	20.69	8.22	20.69	
F4	Before relaxation	15	8.64	2.17	8.40	6.22	11.30	6.22	11.30	*p* = 0.002*
	After relaxation	15	11.74	3.87	12.27	6.96	15.99	6.96	15.99	

*p*, Wilcoxon paired test.

*Statistically significant (*p* < 0.05).

### Alpha, closed eyes

**Figure 2:**
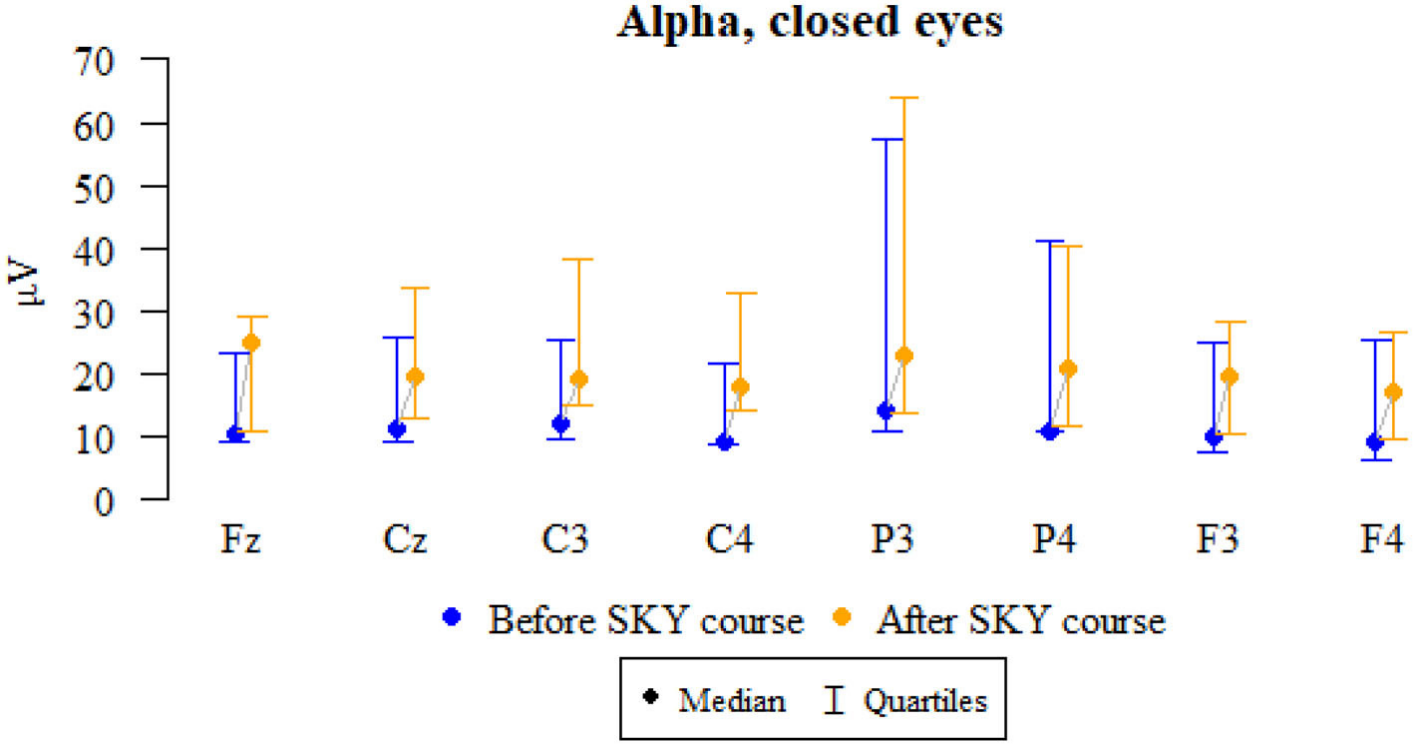
Graphical comparison of Alpha wave amplitudes before the SKY course (the first day) and after the whole SKY course (third day) with eyes closed.

Values of *p* < 0.05 indicate statistically significant relationships:

The amplitude at Fz, Cz, C3, C4, P3, F3, and F4 increased significantly after relaxation exercises (Figure 2 and Table 2).

**Table 2:** Comparison of Alpha wave amplitudes before the SKY course (first day) and after the whole SKY course (third day) with eyes closed.

**Measurement point**	**Measurement**	***N***	**Mean**	**SD**	**Median**	**Min**	**Max**	**Q1**	**Q3**	***p***
Fz	Before relaxation	15	14.35	6.57	10.49	9.35	23.22	9.35	23.22	*p* = 0.002*
	After relaxation	15	21.71	8.12	24.92	11.00	29.21	11.00	29.21	
Cz	Before relaxation	15	15.39	7.64	11.34	9.17	25.66	9.17	25.66	*p* = 0.002*
	After relaxation	15	22.15	9.03	19.74	12.97	33.74	12.97	33.74	
C3	Before relaxation	15	15.60	7.20	12.10	9.48	25.23	9.48	25.23	*p* = 0.002*
	After relaxation	15	24.14	10.69	19.12	14.89	38.41	14.89	38.41	
C4	Before relaxation	15	13.12	6.18	9.13	8.74	21.49	8.74	21.49	*p* = 0.002*
	After relaxation	15	21.73	8.50	17.97	14.20	33.03	14.20	33.03	
P3	Before relaxation	15	27.50	22.06	14.24	10.96	57.31	10.96	57.31	*p* = 0.002*
	After relaxation	15	33.55	22.80	23.03	13.67	63.94	13.67	63.94	
P4	Before relaxation	15	20.98	14.89	11.05	10.74	41.14	10.74	41.14	*p* = 0.321
	After relaxation	15	24.12	12.45	20.66	11.56	40.14	11.56	40.14	
F3	Before relaxation	15	14.25	7.94	10.18	7.67	24.90	7.67	24.90	*p* = 0.002*
	After relaxation	15	19.60	7.66	19.83	10.51	28.46	10.51	28.46	
F4	Before relaxation	15	13.67	8.82	9.31	6.22	25.48	6.22	25.48	*p* = 0.002*
	After relaxation	15	17.79	7.33	17.15	9.53	26.68	9.53	26.68	

*p*, Wilcoxon paired test.

*Statistically significant (*p* < 0.05).

### Beta 1, open eyes

**Figure 3:**
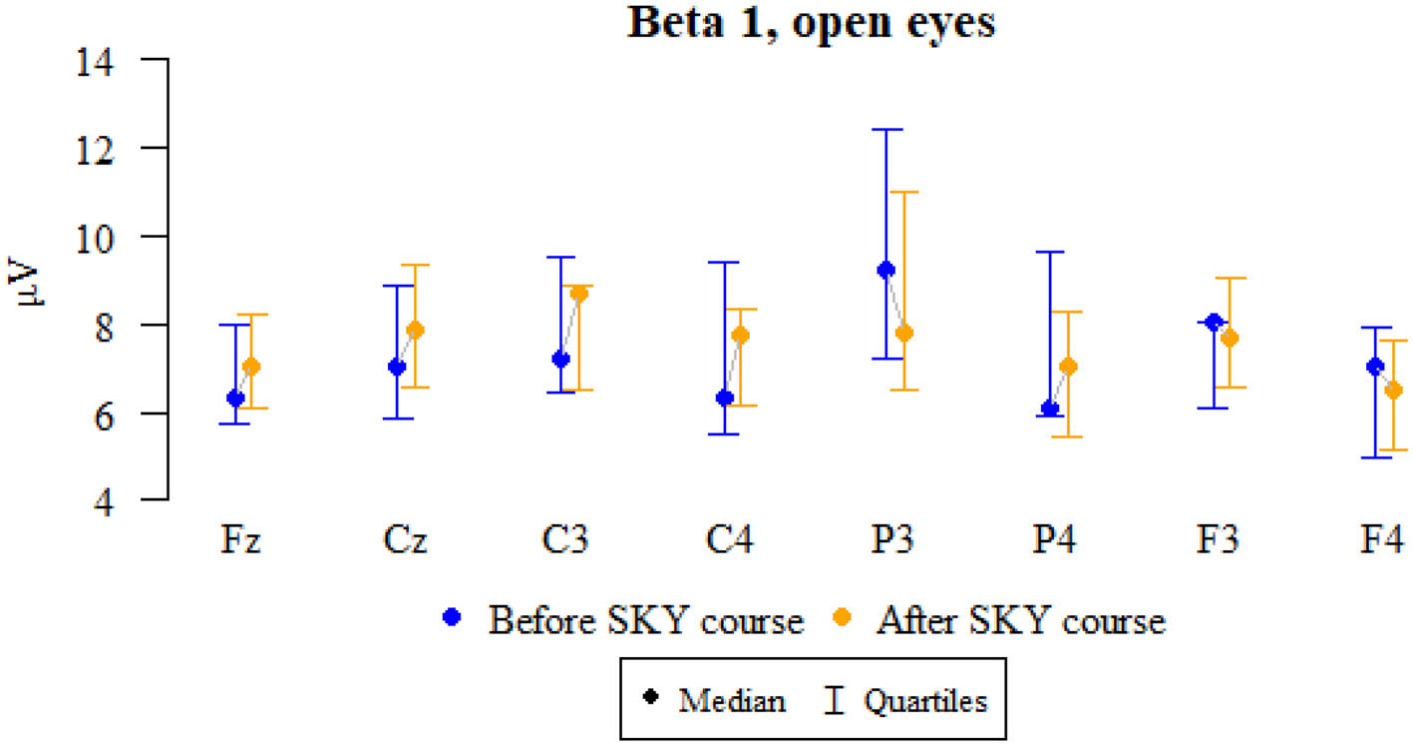
Graphical comparison of Beta 1 wave amplitudes before the SKY course (first day) and after the whole SKY course (third day) with eyes open.

Values of *p* < 0.05 indicate statistically significant relationships:

The amplitude at the P3 and F3 points decreased significantly after the relaxation exercises (Figure 3 and Table 3).

**Table 3:** Comparison of Beta 1 wave amplitudes before the SKY course (the first day) and after the whole SKY course (third day) with eyes open.

**Measurement point**	**Measurement**	***N***	**Mean**	**SD**	**Median**	**Min**	**Max**	**Q1**	**Q3**	***p***
Fz	Before relaxation	15	6.67	1.01	6.30	5.71	7.99	5.71	7.99	*p* = 0.321
	After relaxation	15	7.11	0.92	7.02	6.08	8.23	6.08	8.23	
Cz	Before relaxation	1	7.27	1.30	7.04	5.88	8.90	5.88	8.90	*p* = 0.321
	After relaxation	15	7.91	1.18	7.86	6.55	9.32	6.55	9.32	
C3	Before relaxation	15	7.74	1.37	7.20	6.47	9.54	6.47	9.54	*p* = 0.321
	After relaxation	15	8.01	1.13	8.68	6.49	8.86	6.49	8.86	
C4	Before relaxation	15	7.07	1.74	6.34	5.49	9.38	5.49	9.38	*p* = 0.321
	After relaxation	15	7.42	0.97	7.74	6.16	8.37	6.16	8.37	
P3	Before relaxation	15	9.60	2.24	9.22	7.19	12.40	7.19	12.40	*p* = 0.024*
	After relaxation	15	8.44	1.97	7.80	6.52	11.00	6.52	11.00	
P4	Before relaxation	15	7.22	1.80	6.09	5.91	9.66	5.91	9.66	*p* = 0.321
	After relaxation	15	6.93	1.22	7.03	5.45	8.31	5.45	8.31	
F3	Before relaxation	15	7.40	0.96	8.02	6.10	8.07	6.10	8.07	*p* = 0.024*
	After relaxation	15	7.77	1.05	7.69	6.58	9.03	6.58	9.03	
F4	Before relaxation	15	6.63	1.29	7.02	4.96	7.90	4.96	7.90	*p* = 0.843
	After relaxation	15	6.42	1.08	6.50	5.12	7.64	5.12	7.64	

*p*, Wilcoxon paired test.

*Statistically significant (*p* < 0.05).

### Beta 1, closed eyes

**Figure 4:**
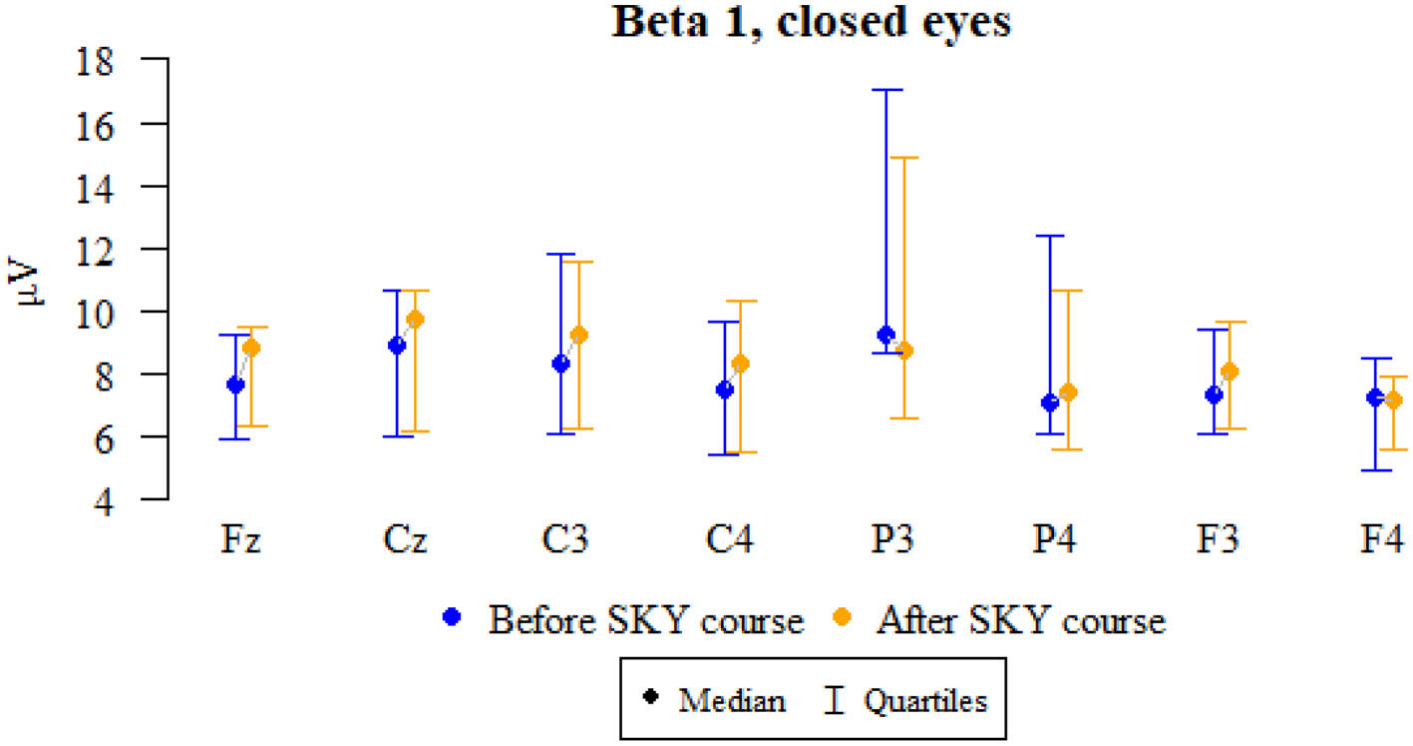
Graphical comparison of Beta 1 wave amplitudes before the SKY course (first day) and after the whole SKY course (third day) with eyes closed.

Values of *p* < 0.05 indicate statistically significant relationships:

— The amplitude at P3 dropped significantly after the relaxation exercises.

— The amplitude at Fz, Cz, C4, P4, and F3 increased significantly after the relaxation exercises (Figure 4 and Table 4).

**Table 4:** Comparison of Beta 1 wave amplitudes before the SKY course (first day) and after the whole SKY course (third day) with eyes closed.

**Measurement point**	**Measurement**	**N**	**Mean**	**SD**	**Median**	**Min**	**Max**	**Q1**	**Q3**	***P***
Fz	Before relaxation	15	7.60	1.41	7.69	5.90	9.21	5.90	9.21	*p* = 0.002*
	After relaxation	15	8.23	1.43	8.83	6.34	9.53	6.34	9.53	
Cz	Before relaxation	15	8.52	2.01	8.91	5.99	10.65	5.99	10.65	*p* = 0.024*
	After relaxation	15	8.84	2.00	9.71	6.18	10.63	6.18	10.63	
C3	Before relaxation	15	8.75	2.46	8.36	6.08	11.80	6.08	11.80	*p* = 0.321
	After relaxation	15	9.01	2.28	9.23	6.23	11.56	6.23	11.56	
C4	Before relaxation	15	7.55	1.81	7.53	5.44	9.68	5.44	9.68	*p* = 0.002*
	After relaxation	15	8.08	2.05	8.33	5.56	10.36	5.56	10.36	
P3	Before relaxation	15	11.67	4.00	9.22	8.71	17.08	8.71	17.08	*p* = 0.024*
	After relaxation	15	10.07	3.67	8.72	6.60	14.88	6.60	14.88	
P4	Before relaxation	15	8.51	2.87	7.09	6.09	12.36	6.09	12.36	*p* = 0.024*
	After relaxation	15	7.90	2.17	7.46	5.61	10.64	5.61	10.64	
F3	Before relaxation	15	7.61	1.43	7.33	6.10	9.41	6.10	9.41	*p* = 0.002*
	After relaxation	15	8.00	1.47	8.09	6.23	9.67	6.23	9.67	
F4	Before relaxation	15	6.89	1.52	7.22	4.96	8.48	4.96	8.48	*p* = 0.843
	After relaxation	15	6.91	1.00	7.15	5.64	7.94	5.64	7.94	

*p*, Wilcoxon paired test.

*Statistically significant (*p* < 0.05).

### Beta 2, open eyes

**Figure 5:**
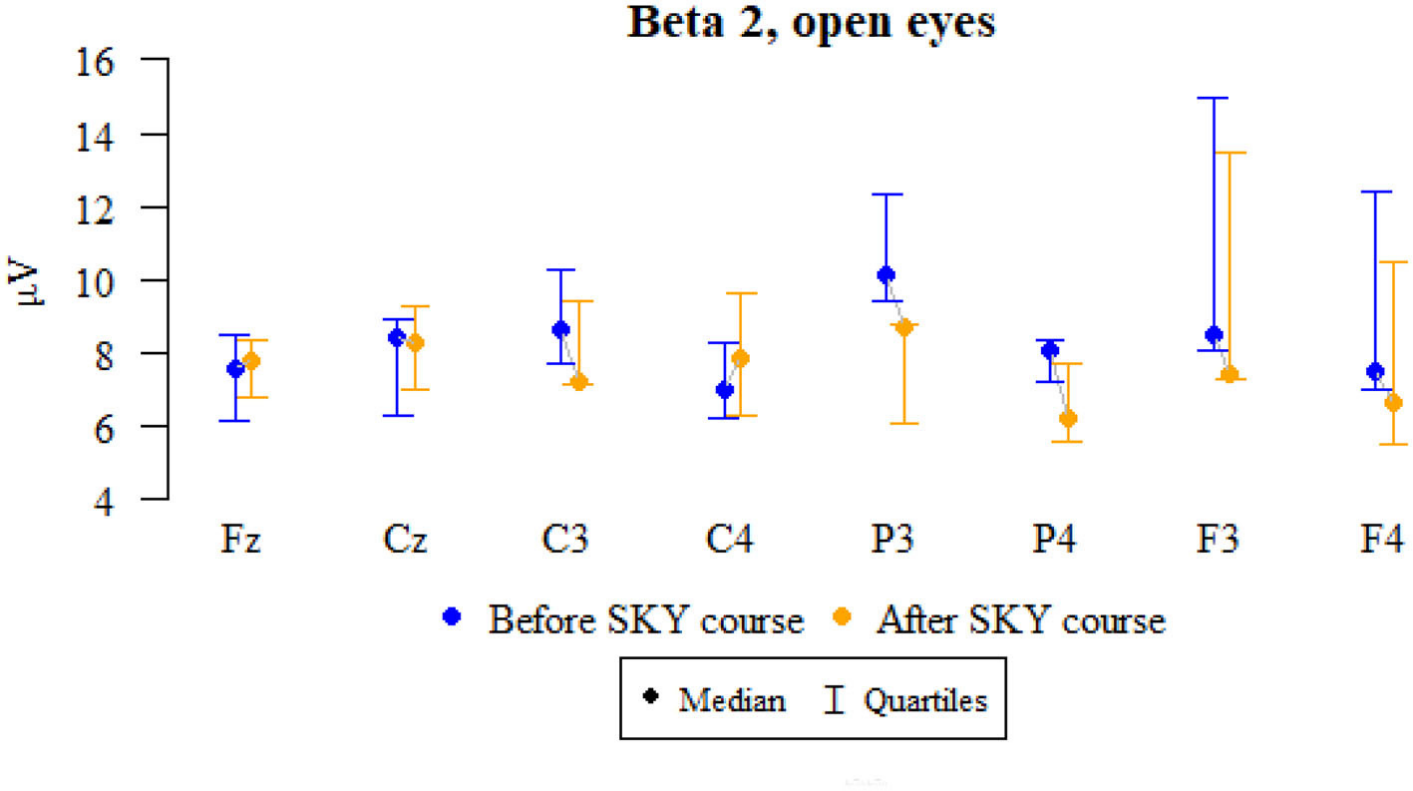
Graphical comparison of Beta 2 wave amplitudes before the SKY course (first day) and after the whole SKY course (third day) with eyes open.

Values of *p* < 0.05 indicate statistically significant relationships:

The amplitude at the P3, P4, F3, and F4 points decreased significantly after the relaxation exercises (Figure 5 and Table 5).

**Table 5:** Comparison of Beta 2 wave amplitudes before the SKY course (first day) and after the whole SKY course (third day) with eyes open.

**Measurement point**	**Measurement**	***N***	**Mean**	**SD**	**Median**	**Min**	**Max**	**Q1**	**Q3**	***p***
Fz	Before relaxation	15	7.40	1.02	7.58	6.13	8.50	6.13	8.50	*p* = 0.843
	After relaxation	15	7.65	0.70	7.79	6.76	8.39	6.76	8.39	
Cz	Before relaxation	15	7.87	1.20	8.41	6.27	8.92	6.27	8.92	*p* = 0.321
	After relaxation	15	8.19	0.99	8.27	6.99	9.31	6.99	9.31	
C3	Before relaxation	15	8.87	1.12	8.62	7.70	10.28	7.70	10.28	*p* = 0.321
	After relaxation	15	7.91	1.10	7.22	7.12	9.40	7.12	9.40	
C4	Before relaxation	15	7.19	0.89	7.00	6.26	8.32	6.26	8.32	*p* = 0.321
	After relaxation	15	7.93	1.44	7.84	6.29	9.67	6.29	9.67	
P3	Before relaxation	15	10.61	1.30	10.10	9.40	12.33	9.40	12.33	*p* = 0.002*
	After relaxation	15	7.86	1.29	8.70	6.12	8.77	6.12	8.77	
P4	Before relaxation	15	7.86	0.51	8.04	7.19	8.34	7.19	8.34	*p* = 0.024*
	After relaxation	15	6.51	0.91	6.24	5.61	7.69	5.61	7.69	
F3	Before relaxation	15	10.52	3.28	8.53	8.07	14.95	8.07	14.95	*p* = 0.002*
	After relaxation	15	9.38	3.00	7.40	7.31	13.44	7.31	13.44	
F4	Before relaxation	15	8.98	2.55	7.52	6.99	12.42	6.99	12.42	*p* = 0.002*
	After relaxation	15	7.56	2.20	6.66	5.55	10.46	5.55	10.46	

*p*, Wilcoxon paired test.

*Statistically significant (*p* < 0.05).

### Beta 2, closed eyes

**Figure 6:**
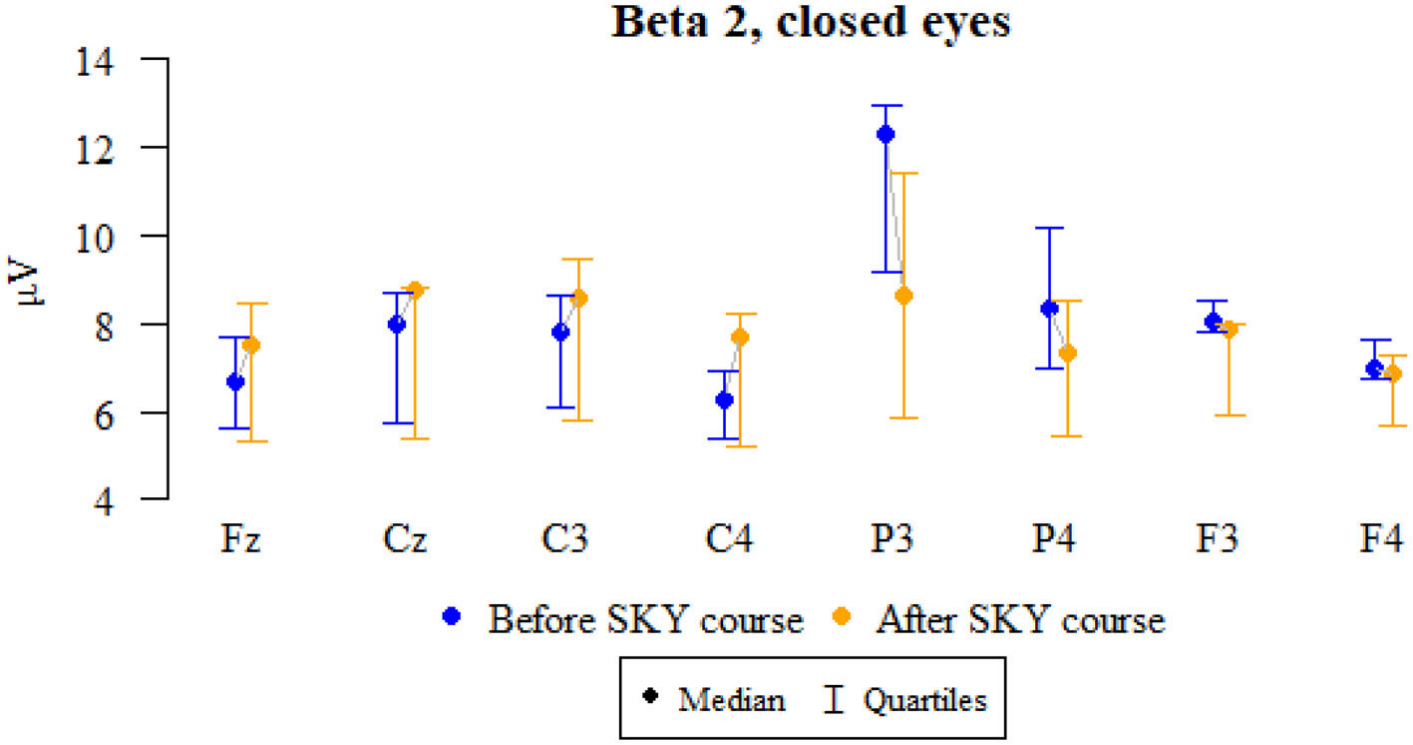
Graphical comparison of Beta 2 wave amplitudes before the SKY course (first day) and after the whole SKY course (third day) with eyes closed.

Values of *p* < 0.05 indicate statistically significant relationships:

The amplitude at the P3, P4, F3, and F4 points decreased significantly after the relaxation exercises (Figure 6 and Table 6).

**Table 6:** Comparison of Beta 2 wave amplitudes before the SKY course (first day) and after the whole SKY course (third day) with eyes closed.

**Measurement point**	**Measurement**	***N***	**Mean**	**SD**	**Median**	**Min**	**Max**	**Q1**	**Q3**	***P***
Fz	Before relaxation	15	6.67	0.90	6.66	5.62	7.72	5.62	7.72	*p* = 0.024*
	After relaxation	15	7.08	1.37	7.50	5.31	8.44	5.31	8.44	
Cz	Before relaxation	15	7.47	1.34	8.00	5.71	8.71	5.71	8.71	*p* = 0.321
	After relaxation	15	7.64	1.68	8.76	5.37	8.79	5.37	8.79	
C3	Before relaxation	15	7.53	1.11	7.82	6.10	8.66	6.10	8.66	*p* = 0.024*
	After relaxation	15	7.95	1.64	8.60	5.79	9.46	5.79	9.46	
C4	Before relaxation	15	6.20	0.67	6.26	5.38	6.95	5.38	6.95	*p* = 0.024*
	After relaxation	15	7.04	1.37	7.71	5.21	8.20	5.21	8.20	
P3	Before relaxation	15	11.49	1.75	12.33	9.15	12.98	9.15	12.98	*p* = 0.002*
	After relaxation	15	8.66	2.38	8.65	5.88	11.45	5.88	11.45	
P4	Before relaxation	15	8.51	1.38	8.34	6.98	10.20	6.98	10.20	*p* = 0.024*
	After relaxation	15	7.10	1.31	7.32	5.47	8.51	5.47	8.51	
F3	Before relaxation	15	8.13	0.31	8.07	7.80	8.51	7.80	8.51	*p* = 0.024*
	After relaxation	15	7.28	0.99	7.88	5.94	8.01	5.94	8.01	
F4	Before relaxation	15	7.12	0.37	6.99	6.76	7.61	6.76	7.61	*p* = 0.024*
	After relaxation	15	6.60	0.72	6.89	5.65	7.25	5.65	7.25	

*p*, Wilcoxon paired test.

*Statistically significant (*p* < 0.05).

The amplitude at Fz, C3, and C4 increased significantly after the relaxation exercises.

## Discussion

This experiment was conducted to determine whether a short-term SKY intervention could improve QEEG parameters in patients who had been infected with the SARS-CoV-2 virus. In this pilot study, we sought to determine the parameters and to what extent they changed under the influence of the applied breathing technique in patients with a history of viral infection, what benefits this may bring, and establish the validity and direction of further research. We found significant differences in the amplitudes of the tested frequencies in the subjects after the applied intervention. A statistically significant increase in the amplitude of the alpha frequency was observed, both with eyes closed and open, when brain waves were recorded. Changes were also noted in relation to Beta frequency, where with eyes open at two reference points, P3 and F3; the amplitude decreased after applying the SKY breathing technique, while with eyes closed, only a decrease in the amplitude at point P3 was noted when there was a significant increase in the amplitude at points Fz, Cz, C4, P4, and F3. Similar observations were made in the recorded Beta 2 frequencies, where with eyes open, there was a decrease in the amplitude at the parietal points P3, P4, and the frontal area, while after closing the eyes, the previously observed decrease remained at the same points, but there was a simultaneous increase in the amplitude at the central points after the SKY breathing technique. This suggests that SKY, after just three interventions, significantly affects brain activity by changing the amplitude of emitted brain waves, thereby improving parameters that translate into the improvement of cognitive abilities such as concentration and memory. This is particularly important because this study was conducted on individuals 2 months after infection with the SARS-CoV-2 virus, which, as mentioned above, negatively affects brain activity, causing impaired concentration and short-term memory, generally characterized by distraction and forgetfulness. Thus, this study provides information that even after infection with a virus that is capable of deteriorating brainwave parameters; however, breathing-based exercises based on the SKY method are able to normalize or even improve them. This pilot study suggests that Alpha, Beta 1, and Beta 2 brain waves have been increased after two SKY sessions, thus increasing the ability to maintain the brain-immune axis to regain homeostasis and return to better functioning. An pilot intervention studies on COVID-19 patients in Milan, Italy, showed promising results in improving lung function and overall patient recovery. The intervention included a 4-min video clip demonstrating a simple breathing practice (Nagendra, 2020). The study indicated pandemic contact with patients with PTSD (posttraumatic stress disorder), which is a common anxiety disorder in response to a traumatic event that has a major impact on mental status. It can cause severe impairment and stress. Experiencing an infectious disease, epidemic, or pandemic such as COVID-19 causes a certain type of physical and mental health problems (Xiao et al., 2020). Other authors have also reported that SKY practice affects brain activity. In a study from 2017 by Chandra S. et al., EEG and ECG results were taken from 25 subjects randomly assigned to the control and experimental groups. The experimental group received the SKY intervention (short/so-called “home SKY”) lasting 30 min for 90 days. The authors' goal was to evaluate the effect of breathing-based meditation on optimizing task performance through stress regulation. Comparisons were made between pre- and post-meditation and experimental groups for theta (4–8 Hz), alpha (8–13 Hz), beta (13–30 Hz), and gamma (30–45 Hz) bands. At rest, alpha and beta energy decreased in the control group, while it increased in the experimental group. This was the most significant post-effect of SKY. The performance of the experimental group improved as their ability to cope with stress increased, which overall increased their workload capacity (Chandra et al., 2017a). Another research paper by Chandra et al., also from 2017, focused on analyzing the effects of SKY on EEG and ECG signals on stress regulation. The subjects were divided into two groups of ten people each. Both groups underwent EEG and ECG testing at the beginning of the study, and then a 30-day SKY session was implemented in the experimental group. The results showed a reduction in alpha band power in the frontal lobe. The authors' observations concluded that the EEG record significantly indicates a reduction in mental stress and an improvement in cognitive performance after SKY (Chandra et al., 2017b). Similarly, the results of the study by Daniel et al. in 2022 showed that the effectiveness of SKY was reflected in improved information processing in the brain. The experimental group showed an improvement in performance regarding the increased beta band complexity for non-linear features, which implies the effective use of cognitive resources during the task (Daniel et al., 2022). Sharma et al. (2019) tested the effects of Sudarshan Kriya Yoga on the EEG and came to the same conclusion, indicating that the SKY breathing technique could possibly be used in clinical practice. Their study aimed to evaluate the effects of SKY on the human brain by means of EEG signals. The EEG signals from 50 subjects were acquired and classified as meditating or non-meditating based on statistical parameters. Pre- and post-electroencephalogram (EEG) signals were collected from control and test groups before and after 3 months of regular SKY practice. Their results showed that the values of variance, standard deviation, zero crossing, and maximum and minimum of EEG signals increased in the study group but decreased in the control group (Sharma et al., 2019). The study by Srinivasan and Baijal (2007) examined the effects of SKY practice on the Mismatch Negativity Paradigm (MMN), an indicator of preattentive processing. Auditory MMN waveforms were recorded at the beginning and after each practice in the meditators and similarly after the relaxation sessions in non-meditators. Meditators have been found to have higher MMN amplitudes than non-meditators. Meditators also showed significantly increased MMN amplitudes immediately after meditation, suggesting transient state changes due to meditation. The results indicate that SKY practice enhances preattentive perceptual processes, enabling better detection of changes in auditory sensory memory (Srinivasan and Baijal, 2007). Bhaskar et al. (2020) conducted a study in which they observed changes in the brain with EEG after the SKY breathing technique. They showed a significant increase in frequency in all brain waves, bilaterally, after SKY. They also showed an asymmetry index between inter-hemispheric synchronization and SKY practice, which was around 0 after the SKY practice. The researchers concluded that even a single SKY session could generate a global brain rhythm dominated by high-frequency brain activation and initiates appropriate inter-hemispheric synchronization (Bhaskar et al., 2020). This suggests that SKY leads to improved attention, memory, emotional, and autonomic control, along with enhanced cognitive function, ultimately improving physical and mental wellbeing. So far, scientific reports examining the effects of SKY on brainwaves are very limited and relatively new to the scientific world. To date, no scientific studies have examined the effects of SKY on brainwave parameters in people who have suffered from COVID-19. Most existing studies are based on comparisons of EEG results in people with and without SKY intervention. They differ with respect to long SKY (performed during courses, maximum once a week, ~1.5 h), short/home SKY (30 min, every day), the number of examined days, and the cognitive abilities and their changes due to the SKY practice. However, most of the existing studies, like this one, indicate that SKY positively affects brain activity. In analyzing existing studies, the first limitation appears in their small number. To be able to relate the results of our study to those that already exist, to compare them, and more accurately find and describe differences and similarities. Moreover, there are no controversies or side effects to practicing this technique. The technique itself can be cost-free, home-based prophylaxis. Even though our study was conducted on a small group of subjects, it provides a qualitative basis for further research. Due to the timeliness of our work, it provides a great example for other researchers. Both the studies conducted by other researchers and by us certainly indicate a positive effect of practicing SKY on the improvement of QEEG parameters and, therefore, on overall brain activity.

## Conclusions

Our study provides significant insight into the effects of SKY practice on brain activity. After just one 3-day course of SKY, the QEEG study shows changes in the amplitude of the brain waves related to the state of relaxation and concentration, indicating their statistically significant improvement. These results are, first of all, an important fact, demonstrating that SKY influences the state of relaxation and significantly improves concentration, which is of particular importance in post-COVID-19 patients due to post-COVID concentration disorders that have been demonstrated in numerous scientific studies. Secondly, this study provides enough relevant information to conclude that practicing SKY might be recommended in the face of a pandemic. Third, this study justifies the need for further research in this direction. The current study has limitations, such as a small number of subjects. Nevertheless, it has shown changes in the results, which is a strong suggestion for further research on a larger group of people. This could enhance the understanding of changes in QEEG recordings influenced by practicing SKY.

## Limitations

Our study, like many others, has limitations. The most crucial factor is the small number of study participants. The SKY technique is not that ubiquitous in Poland, and some people may be skeptical about this method. Age was another limitation. Participating patients belong to a similar age range to avoid age differences affecting the body's condition. The study included people from one geographical area, which positively influenced the lack of climate change, but the variety of virus mutations and the possibility to distinguish its variants in a larger number of responders. Nevertheless, our research shows clear differences in QEEG recording before and after the breathing practice. We believe these results provide a good basis for further QEEG-based research on the SKY technique due to the globally increasing statistics on the long-term effects of COVID-19.

## Data availability statement

The raw data supporting the conclusions of this article will be made available by the authors, without undue reservation.

## Ethics statement

The study was conducted in accordance with the Declaration of Helsinki, and approved by the Ethics Committee of University of Rzeszow (protocol code 8/12/2021). The patients/participants provided their written informed consent to participate in this study.

## Author contributions

MK and AB-Z: conceptualization, methodology, and investigation. RM: software and validation. WM, AŁ, and BK: formal analysis and writing-review and editing. MK, AŁ, WM, and BK: writing-original draft preparation. All authors have read and agreed to the published version of the manuscript.

## Conflict of interest

The authors declare that the research was conducted in the absence of any commercial or financial relationships that could be construed as a potential conflict of interest.

## Publisher's note

All claims expressed in this article are solely those of the authors and do not necessarily represent those of their affiliated organizations, or those of the publisher, the editors and the reviewers. Any product that may be evaluated in this article, or claim that may be made by its manufacturer, is not guaranteed or endorsed by the publisher.
